# Complete genome sequence of a *Circovirus pigeon* strain in lymphocyte-depleted bursa of Fabricius of a Common Raven (*Corvus corax*)

**DOI:** 10.1128/mra.00378-24

**Published:** 2024-08-30

**Authors:** Marie-Jeanne Pesant, Shannon Ferrell, Marika Köszegi, Vincent Baby, Valérie Grenier Saint-Sauveur, Camila Valle Tejada, Stéphane Lair, Carl A. Gagnon

**Affiliations:** 1 Swine and Poultry Infectious Diseases Research Center (CRIPA-FRQ), Faculté de médecine vétérinaire, Université de Montréal, Saint-Hyacinthe, Québec, Canada; 2 Centre de diagnostic vétérinaire de l’Université de Montréal (CDVUM), Faculté de médecine vétérinaire, Université de Montréal, Saint-Hyacinthe, Québec, Canada; 3 Centre québécois sur la santé des animaux sauvages (CQSAS) - Canadian Wildlife Health Cooperative, Faculté de médecine vétérinaire, Université de Montréal, Saint-Hyacinthe, Québec, Canada; Katholieke Universiteit Leuven, Leuven, Belgium

**Keywords:** circovirus, viral complete genome, pigeon circovirus, common raven

## Abstract

A necropsy was performed on a Common Raven (*Corvus corax*) presenting an opportunistic fungal respiratory infection and a bursal lymphoid depletion with inclusion bodies, suggestive of a circovirus infection. High-throughput sequencing of circular DNA in the bursa of Fabricius revealed a complete genome sequence of a *Circovirus pigeon* strain.

## ANNOUNCEMENT

Circoviruses (family *Circoviridae*, genus *Circovirus*) are small, non-enveloped viruses that contain a circular, single-stranded DNA genome and can infect a wide spectrum of avian hosts (1). Many cases of avian circovirus natural spillover infections in aberrant hosts, such as *Circovirus pigeon* (PiCV) in ducks or magpies, have been reported (2). PiCV, first diagnosed in 1993 (3) and recognized as a species in 2005 (4), is considered one of the most important pathogens affecting pigeon health (5). Some other avian circovirus species, such as *Circovirus raven* (RaCV), are poorly characterized, which is represented by only one known complete genome sequence identified in an Australian Raven (*Corvus coronoides*) in 2006 (6).

Through wildlife health surveillance activities of the CQSAS, a necropsy was performed on a Common Raven (*Corvus corax*) found dead in Saint-Jérôme (Québec, Canada) in 2022. Routine histopathology revealed that the emaciated bird presented an opportunistic fungal respiratory infection in its air sacs, as well as a bursal lymphoid depletion with inclusion bodies, observed *via* Hematoxylin & Eosin staining, suggestive of a viral infection (7). It is well known that circoviruses cause lymphocyte depletion with subsequent immunosuppression in several species of mammals and birds (8).

Upon negative PCR results testing for highly pathogenic H5N1 avian influenza (9, 10), West Nile virus (11), and chicken anemia virus (12), next-generation sequencing for an underlying circovirus infection was performed with extracted viral DNA (*Quick*-Viral DNA, Zymo) from the raven’s bursa of Fabricius. Circular DNA was amplified using the Rolling Circle Amplification (RCA) technique with ThermoFisher’s EquiPhi29 enzyme as recommended, and the sequencing library was prepared using Illumina’s Nextera XT kit as recommended by the manufacturer. Sequencing was carried out with a 600-cycle-v3 (300 base reads) on Illumina’s MiSeq system followed by read quality assessment, read trimming, host genome depletion and assemblies using CLC Genomics Workbench v24.0 (QIAGEN), and genome annotation using Geneious Prime v2022.1 (Dotmatics) both using default parameters. A total of 2,738,764 reads were obtained and were analyzed by *de novo* and reference-based (PiCV; NC_002361) assembly. These included 633,793 PiCV-specific reads, which revealed a 2,043 nucleotide-long circovirus genome, with an average coverage of 93,068× and a GC content of 55.05%. An in-house qPCR assay, targeting a conserved region of the REP gene, was developed. Primers and probe were designed as follows: Forward: 5′-GTTCTACTTACCCTGGGCATTC-3′; Reverse: 5′-CGTATATCTCACTGAAGTCTCGC-3′; Probe: 5'-(6FAM)CGGTCATTGCTCTTCCGGCTTTCA(MBG-NFQ)-3*'*. The TaqMan Fast Advanced MasterMix was used in this qPCR assay as recommended by the manufacturer (ThermoFisher Scientific). It confirmed the presence of PiCV in 40 ng of total extracted DNA from the bursa of the Fabricius sample (Ct_mean_ ± Ct_SD_ = 30.97 ± 0.10).

**Fig 1:**
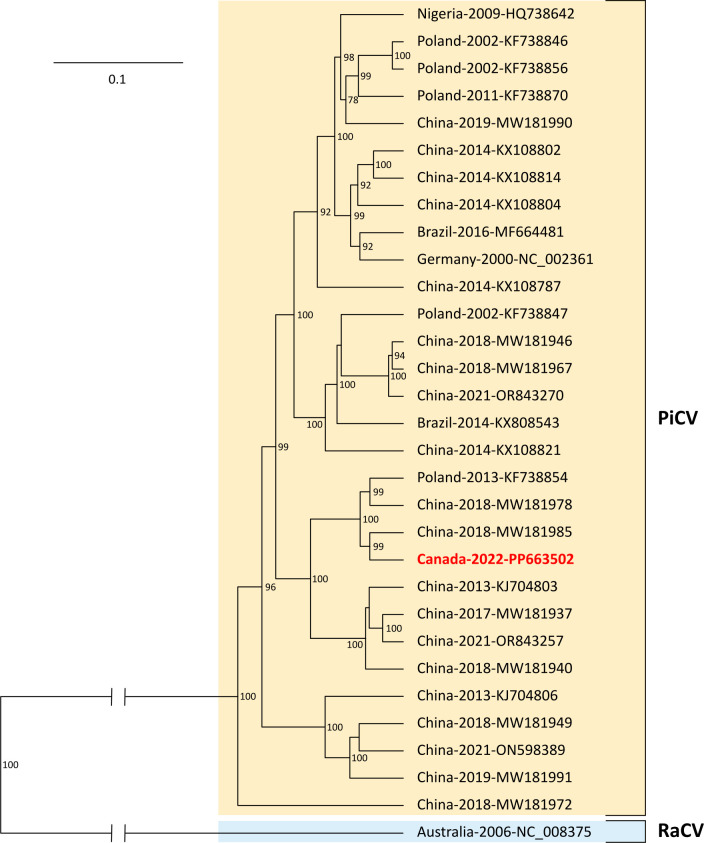
Posterior maximum clade credibility tree of *Circovirus pigeon* (PiCV) and *Circovirus raven* (RaCV) complete genomes. The whole viral genome nucleotide sequences were aligned using Clustal Omega (v1.2.4) (13) with default parameters, and poorly aligned positions were removed using Gblocks (v0.91b) (15) resulting in a final alignment of 1741 nucleotide sites. The Bayesian inference phylogenetic tree was made using BEAST (v2.7.7) (16) and its support programs including Beauti to set up the analysis and TreeAnnotator to generate the final tree, default parameters were used unless specified. The SYM+ gamma4 substitution model was selected with estimation of the proportion of invariable sites and the coalescent prior with exponential population growth was used. The substitution model for the tree building was identified using ModelFinder (17) on the W-IQ-TREE web server (v1.6.12) (18). Tree visualization was made using FigTree (v1.4.4; available at https://github.com/rambaut/figtree/releases). Sequences are identified in the phylogenetic tree as follows: “Country of origin”-“Year”-“GenBank Accession #” (in red and bold for the new sequence of interest). Posterior probabilities are indicated in percentage on each node and values <70% were removed.

Clustal Omega (v1.2.4) (13) was used to determine the nucleotide (nt) identity between the newly acquired sequence, RaCV, and other PiCVs found in the literature. The nt identity between the new sequence and PiCVs varied from 81.50% to 95.64% illustrating that the new sequence is of the same species based on the 80% cutoff required to denote a different species within the *Circoviridae* family (14). The nt identity between the new sequence and the RaCV whole genome was 48.89%, revealing that both viruses found in ravens are, as clearly supported by positions in the phylogenetic tree (see Fig. 1), two different species of the *Circoviridae* family.

Overall, these findings demonstrate the first complete genome sequence of a circovirus identified from a Common Raven and highlight a new susceptible host for the *Circovirus pigeon* viral species. This could have speculatively originated from a spillover through overlapping dietary and habitat niches of the two avian hosts.

## Data Availability

The high-throughput sequencing data are available in the SRA database under the BioProject no. PRJNA1096031, and the complete genome sequence of the Common raven pigeon-like circovirus strain CDVUM 2807247 has been deposited in GenBank under accession no. PP663502. The version described in this paper is the first version, PP663502.1.

## References

[1] Todd D, McNulty MS, Adair BM, Allan GM. 2001. Animal circoviruses, p 1–70. In Advances in virus research. Academic Press. 10.1016/s0065-3527(01)57000-1 11680382

[2] Nath BK, Das T, Peters A, Gupta SD, Sarker S, Forwood JK, Raidal SR, Das S. 2023. Australasian pigeon circoviruses demonstrate natural spillover infection. Viruses 15:2025. doi:10.3390/v15102025 37896802 PMC10611180

[3] Woods LW, Latimer KS, Barr BC, Niagro FD, Campagnoli RP, Nordhausen RW, Castro AE. 1993. Circovirus-like infection in a pigeon. J Vet Diagn Invest 5:609–612. doi:10.1177/104063879300500417 8286462

[4] Fauquet CM, Fargette D. 2005. International committee on taxonomy of viruses and the 3,142 unassigned species. Virol J 2:64. doi:10.1186/1743-422X-2-64 16105179 PMC1208960

[5] Silva BBI, Urzo MLR, Encabo JR, Simbulan AM, Lunaria AJD, Sedano SA, Hsu K-C, Chen C-C, Tyan Y-C, Chuang K-P. 2022. Pigeon circovirus over three decades of research: bibliometrics, scoping review, and perspectives. Viruses 14:1498. doi:10.3390/v14071498 35891478 PMC9317399

[6] Stewart ME, Perry R, Raidal SR. 2006. Identification of a novel circovirus in Australian ravens (Corvus coronoides) with feather disease. Avian Pathol 35:86–92. doi:10.1080/03079450600597345 16595298

[7] Maclachlan NJ, Dubovi EJ. 2017. Circoviridae and anelloviridae, p 259–268. In Fenner’s veterinary virology, 5th ed

[8] Fehér E, Jakab F, Bányai K. 2023. Mechanisms of circovirus immunosuppression and pathogenesis with a focus on porcine circovirus 2: a review. Vet Q 43:1–18. doi:10.1080/01652176.2023.2234430 PMC10367577 37431709

[9] Weingartl HM, Berhane Y, Hisanaga T, Neufeld J, Kehler H, Emburry-Hyatt C, Hooper-McGreevy K, Kasloff S, Dalman B, Bystrom J, Alexandersen S, Li Y, Pasick J. 2010. Genetic and pathobiologic characterization of pandemic H1N1 2009 influenza viruses from a naturally infected swine herd. J Virol 84:2245–2256. doi:10.1128/JVI.02118-09 20015998 PMC2820904

[10] Spackman E, Senne DA, Myers TJ, Bulaga LL, Garber LP, Perdue ML, Lohman K, Daum LT, Suarez DL. 2002. Development of a real-time reverse transcriptase PCR assay for type A influenza virus and the avian H5 and H7 hemagglutinin subtypes. J Clin Microbiol 40:3256–3260. doi:10.1128/JCM.40.9.3256-3260.2002 12202562 PMC130722

[11] Lanciotti RS, Kerst AJ, Nasci RS, Godsey MS, Mitchell CJ, Savage HM, Komar N, Panella NA, Allen BC, Volpe KE, Davis BS, Roehrig JT. 2000. Rapid detection of West Nile virus from human clinical specimens, field-collected mosquitoes, and avian samples by a TaqMan reverse transcriptase-PCR assay. J Clin Microbiol 38:4066–4071. doi:10.1128/JCM.38.11.4066-4071.2000 11060069 PMC87542

[12] Imai K, Mase M, Yamaguchi S, Yuasa N, Nakamura K. 1998. Detection of chicken anaemia virus DNA from formalin-fixed tissues by polymerase chain reaction. Res Vet Sci 64:205–208. doi:10.1016/s0034-5288(98)90126-6 9690604

[13] Sievers F, Wilm A, Dineen D, Gibson TJ, Karplus K, Li W, Lopez R, McWilliam H, Remmert M, Söding J, Thompson JD, Higgins DG. 2011. Fast, scalable generation of high-quality protein multiple sequence alignments using clustal Omega. Mol Syst Biol 7:539. doi:10.1038/msb.2011.75 21988835 PMC3261699

[14] Rosario K, Breitbart M, Harrach B, Segalés J, Delwart E, Biagini P, Varsani A. 2017. Revisiting the taxonomy of the family Circoviridae: establishment of the genus Cyclovirus and removal of the genus Gyrovirus. Arch Virol 162:1447–1463. doi:10.1007/s00705-017-3247-y 28155197

[15] Castresana J. 2000. Selection of conserved blocks from multiple alignments for their use in phylogenetic analysis. Mol Biol Evol 17:540–552. doi:10.1093/oxfordjournals.molbev.a026334 10742046

[16] Bouckaert R, Vaughan TG, Barido-Sottani J, Duchêne S, Fourment M, Gavryushkina A, Heled J, Jones G, Kühnert D, De Maio N, Matschiner M, Mendes FK, Müller NF, Ogilvie HA, du Plessis L, Popinga A, Rambaut A, Rasmussen D, Siveroni I, Suchard MA, Wu C-H, Xie D, Zhang C, Stadler T, Drummond AJ. 2019. BEAST 2.5: an advanced software platform for Bayesian evolutionary analysis. PLOS Comput Biol 15:e1006650. doi:10.1371/journal.pcbi.1006650 30958812 PMC6472827

[17] Kalyaanamoorthy S, Minh BQ, Wong TKF, von Haeseler A, Jermiin LS. 2017. ModelFinder: fast model selection for accurate phylogenetic estimates. Nat Methods 14:587–589. doi:10.1038/nmeth.4285 28481363 PMC5453245

[18] Trifinopoulos J, Nguyen L-T, von Haeseler A, Minh BQ. 2016. W-IQ-TREE: a fast online phylogenetic tool for maximum likelihood analysis. Nucleic Acids Res 44:W232–W235. doi:10.1093/nar/gkw256 27084950 PMC4987875

